# GGRaSP: a R-package for selecting representative genomes using Gaussian mixture models

**DOI:** 10.1093/bioinformatics/bty300

**Published:** 2018-04-14

**Authors:** Thomas H Clarke, Lauren M Brinkac, Granger Sutton, Derrick E Fouts

**Affiliations:** 1 J. Craig Venter Institute, Rockville, MD, USA; 2 Department of Biotechnology and Food Technology, Durban University of Technology, Durban, South Africa

## Abstract

**Motivation:**

The vast number of available sequenced bacterial genomes occasionally exceeds the facilities of comparative genomic methods or is dominated by a single outbreak strain, and thus a diverse and representative subset is required. Generation of the reduced subset currently requires a priori supervised clustering and sequence-only selection of medoid genomic sequences, independent of any additional genome metrics or strain attributes.

**Results:**

The Gaussian Genome Representative Selector with Prioritization (*GGRaSP*) R-package described below generates a reduced subset of genomes that prioritizes maintaining genomes of interest to the user as well as minimizing the loss of genetic variation. The package also allows for unsupervised clustering by modeling the genomic relationships using a Gaussian mixture model to select an appropriate cluster threshold. We demonstrate the capabilities of *GGRaSP* by generating a reduced list of 315 genomes from a genomic dataset of 4600 *Escherichia coli* genomes, prioritizing selection by type strain and by genome completeness.

**Availability and implementaion:**

*GGRaSP* is available at https://github.com/JCVenterInstitute/ggrasp/.

**Supplementary information:**

Supplementary data are available at *Bioinformatics* online.

## 1 Introduction

Next-generation sequencing technologies have resulted in a large number of publicly available microbial genome sequences. The number of genomes available for comparative genomic analysis can exceed what can be feasibly visualized or analyzed (Chan *et al.*, 2015; Chavda *et al.*, 2016; Zaslavsky *et al.*, 2016). Additionally, sequencing of clonal or nearly clonal bacterial pathogens involved in disease outbreaks (e.g. *Acinetobacter baumannii*, *Escherichia coli* and *Klebsiella pneumoniae*) can skew the analyses; therefore, a reduction in genome redundancy to maximize diversity is necessary (Chan *et al.*, 2015). One common method to reduce sequence redundancy while minimizing information loss is to cluster genomes by their nucleotide distance metrices and from each cluster select one genome, often a medoid (the genomes with the minimal combined distance to the other genomes in the cluster) (Chan *et al.*, 2015; Moreno-Hagelsieb *et al.*, 2013), as a representative. However, these methods require the user to a priori specify either the number of clusters or a distance cutoff, and they do not allow the user to use the highest quality (i.e. most complete) representative genome for each cluster. Likewise, no dedicated program exists for loading and selecting these genomes.

## 2 Materials and methods

Here, we introduce *GGRaSP* (Gaussian Genome Representative Selector with Prioritization), a R-package and associated executable Rscript program that generates a list of prioritized representative genomes from either supervised or unsupervised clustering of related genomes. *GGRaSP* supports three forms of input to describe the relationship between the genomes: (i) a phylogeny in Newick format; (ii) a distance or similarity matrix; or (iii) an aligned multiple FASTA file. *GGRaSP* uses hierarchical clustering in the hclust R function or the *APE* R-package to create phylogenies from (ii) and (iii) (Paradis *et al.*, 2004). By default, *GGRaSP* prioritizes medoids as representative genomes in order to minimize the loss of information, but this can result in removal of genomes that contain regions of interests (e.g. plasmids, antibiotic resistance islands, pathogenicity islands and prophage), have a more complete assembly, or are from a given project. Users can therefore specify criteria of genomes for selection as representatives by generating a text file containing tiered ranks of the genomes.

*GGRaSP* can cluster genomes using supervised methods, including specifying the number of clusters or the cluster cut-off distance, but it also allows for unsupervised clustering by using Gaussian mixture models (GMMs) to identify a cut-off value that separates the most closely related genomes from the more diverse genomes. GMMs of sequence distances have previously been used to model the evolutionary relationship between multiple genomes in metagenomes (e.g. Alneberg *et al.*, 2014; Ji *et al.*, 2017), and to model homologs descending from distinct ancient large-scale duplications in various eukaryotic organisms (e.g. Cui *et al.*, 2006; Schwager *et al.*, 2017). The GMM model could be biased or limited by collections of genomes which contain a single branch of highly related genomes (for which *GGRaSP* will select a cutoff that will only cluster that single branch) or a set of genomes that can be best modeled by a single Gaussian peak (in which case *GGRaSP* cannot find a cutoff).

**Fig. 1.**
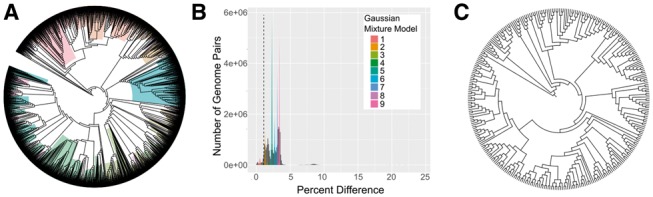
*GGRaSP* based reduction of 4600 *E.coli* genomes 4600 *E.coli* genomes were downloaded from NCBI RefSeq (**A**), clustered using a cut-off (shown as dotted line) determined by GMM (**B**), and reduced to 315 representative genomes (**C**). The colored branches in (A) denote branches reduced to a single node in (C)

In *GGRaSP*, GMMs are calculated using expectation maximization via *mixtools* or *bgmm* (Benaglia *et al.*, 2009; Biecek *et al.*, 2012). Multiple Gaussian distributions are tested incrementally until the addition of a distribution is not significant by the Likelihood Ratio test or exceeds the user defined limit. After the GMM is cleaned by removing overlapping and low count distributions, the inflection point between the first two distributions is used as the cut-off to generate the clusters (see dotted vertical line, Fig. 1). The default pipeline behavior is described earlier, but many of the parameters for the GMM-based threshold calculation are user-modifiable for the cases where the GMM varies from the default model.

*GGRaSP* can output multiple supporting files as is described in detail on the R help pages including: tab-delineated files with information on the clusters; *ggplot2*-based images showing the GMM, the initial or the final phylogenies (Wickham, 2009) with *colorspace* to determine the hues of GMM and phylogeny shading (Ihaka *et al.*, 2016); the Newick files for any phylogeny used in *GGRaSP*; and the iTOL-formatted text files showing the clusters on the phylogenies (Letunic and Bork, 2016). A Rscript version of *GGRaSP* to run on a command line to facilitate high-throughput analyses is also provided.

## 3 Usage scenario

To demonstrate the usefulness of *GGRaSP*, we downloaded 4600 *Escherichia* genomes from NCBI RefSeq on 2/2/2017 using the downloader script in the LOCUST package (Brinkac *et al.*, 2017). A whole genome-based Average Nucleotide Identity (gANI) matrix was calculated with Mash (Ondov *et al.*, 2016). The genomes were ranked, in order by: whether it was a type strain; whether it was circular; and whether it was complete. The remaining genomes were ranked by the number of contigs and genes according to the LOCUST downloader output. The similarity matrix and the ranking file were input to *GGRaSP*, which computed a cut-off of 1.09% identity after modeling 9 Gaussian distributions (10 before clean-up), leading to a selection of 315 representative genomes in 98 min and 2s (Fig. 1, Supplementary Fig. S1). When using to a priori cutoff of 96.5% gANI cutoff suggested for species (Varghese *et al.*, 2015), only nine clusters were generated with the largest cluster containing 98.9% of the genomes. Ranking the genomes as described earlier increased the completeness of retained genomes compared to selecting the representatives from an unranked set number of complete genomes (from 6.7 to 25.4%) and mean N50 (from 205 to 556 kb). All input and output files for these runs and the a priori cutoffs are available on the GitHub repository.

## 4 Conclusion

As the number of sequenced genomes available for comparative genomic analysis continues to expand, the need to generate robust representative genomic subsets will increase. Building off the statistical, bioinformatic, and graphical capabilities of R, *GGRaSP* and the accompanying Rscript provides a single and customizable platform to run multiple analyses to generate a subset of representative genomes. The user can specify clustering parameters and levels of importance for ranking the genomes, thus allowing for both generalizable high-throughput and more dataset specific use.

## Supplementary Material

Supplementary Data
