# Supplementary material for: GGRaSP: a R-package for selecting representative genomes using Gaussian mixture models
Source: Bioinformatics. 2018 Apr 14;34(17):3032–4. doi: 10.1093/bioinformatics/bty300 (PMC6129299; doi:10.1093/bioinformatics/bty300)
Supplement: Supplementary Data [file bty300_supplemental_figure.docx]

GGRaSP: A R-package for selecting representative genomes using Gaussian mixture models

**Supplemental Figure**


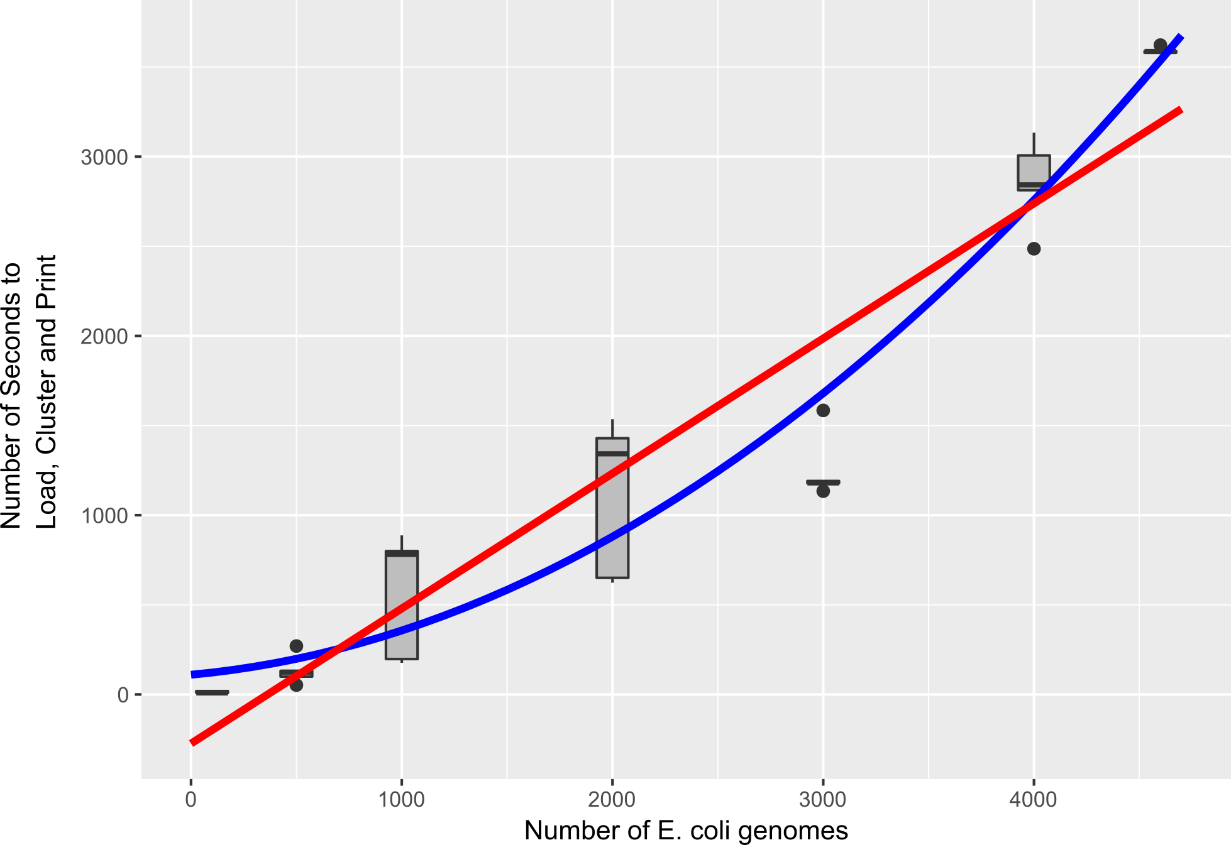


Figure S1. **Time usage for GGRaSP**. GGRaSP runs of 100, 500, 1,000, 2,000, 3,000, or 4,000 randomly sampled genomes from the *E. coli* set was used to estimate time usage. Best-fit linear (red; r^2^ = 0.90) and polynomial (blue; r^2^ = 0.94) models were calculated and are shown.
